# Global Calibration of a Collaborative Multi-Line-Scan Camera Measurement System

**DOI:** 10.3390/s26082498

**Published:** 2026-04-17

**Authors:** Yuanshen Xie, Nanhui Wu, Yueqiao Hou, Weixin Xu, Jiangjie Yu, Zichao Yin, Dapeng Tan

**Affiliations:** 1 College of Mechanical Engineering, Zhejiang University of Technology, Hangzhou 310014, China; nanhuiwu@zjut.edu.cn (N.W.); houyueqiao@zjut.edu.cn (Y.H.); weixinxu@zjut.edu.cn (W.X.); 221122020414@zjut.edu.cn (J.Y.); yingzichao@zjut.edu.cn (Z.Y.); 2 Quzhou Eco-Industrial Innovation Institute ZJUT, Quzhou 324499, China

**Keywords:** multi-line-scan camera system, global calibration, three-dimensional measurement, geometric constraints, hierarchical optimization

## Abstract

**Highlights:**

**What are the main findings?**

- A physics-constrained global bundle adjustment framework is developed for collaborative multi-line-scan camera systems to mitigate scale ambiguity and strong parameter coupling.
- Parameter scaling with a two-stage Levenberg–Marquardt optimization improves convergence reliability, achieving ≥99.7% success under large focal-length initialization perturbations and sub-millimeter 3D reconstruction error in simulation.

**What are the implications of the main findings?**

- The proposed calibration strategy reduces uncertainty and improves numerical stability for multi-line-scan systems in dynamic, multi-camera scenarios, supporting consistent system-level parameter estimation. Provides practical guidance for engineering deployment of high-precision online 3D measurement and dynamic reconstruction.

**Abstract:**

Multi-line-scan camera systems provide high-frequency sampling and wide field-of-view coverage, making them valuable for three-dimensional measurement and dynamic reconstruction. However, their one-dimensional projection property introduces scale ambiguity and strong parameter coupling during calibration, which limits the consistency and stability of local optimization in multi-camera systems. To address this issue, this paper proposes a global calibration method based on physical constraints and hierarchical optimization. A unified imaging and motion model is constructed by incorporating physical scale constraints and structural priors, and geometric scale information is introduced into the joint optimization to reduce scale ambiguity and parameter coupling. Parameter normalization and staged optimization are further adopted to improve numerical stability for variables of different magnitudes and enable consistent estimation of multi-camera parameters within a unified framework. Simulation and experimental results show that the method achieves stable convergence under focal-length initialization perturbation, baseline deviation, and noise interference, with a three-dimensional reconstruction error below 0.67 mm and a convergence probability of at least 99.7%. These results indicate that the proposed method effectively reduces calibration uncertainty in multi-line-scan camera systems and supports high-precision online measurement and dynamic three-dimensional perception.

## 1. Introduction

Line-scan camera measurement systems offer high-frequency sampling, high resolution, and a compact structure, and they have been widely used in dynamic measurement, industrial online inspection, and three-dimensional reconstruction in complex scenes [1,2,3,4,5,6,7]. The calibration accuracy of these systems directly affects spatial measurement results and pose estimation performance. Unlike area-scan cameras that use a two-dimensional imaging plane, line-scan cameras provide only one-dimensional projection information. As a result, a single frame does not provide complete geometric constraints, and scale determination and parameter estimation inherently suffer from insufficient constraints. This limitation becomes more severe in multi-camera collaborative systems and dynamic push-broom imaging, where parameter coupling and scale uncertainty can further accumulate and propagate. Consequently, it becomes more difficult to ensure system consistency and numerical stability. Therefore, developing a stable and accurate global calibration method for complex operating conditions is a key step toward improving the overall performance of line-scan camera measurement systems.

To address the fact that line-scan cameras acquire only one-dimensional image information and therefore cannot directly use conventional calibration models for area-scan cameras, existing studies have mainly developed two technical routes: static calibration and dynamic calibration. Under conditions without push-broom motion, static calibration methods usually establish the mapping between one-dimensional image points and three-dimensional spatial points based on projective geometry and cross-ratio invariance. For target design and parameter estimation under weak constraints, Luna [8], Li [9,10], Lilienblum [11,12], and Song [13] proposed several simple target schemes. These methods can estimate parameters from a small number of static images and reduce dependence on precision motion platforms and complex three-dimensional targets. To improve feature extraction accuracy and suppress errors, Niu et al. [14] used a spatial ring-group target and combined subpixel edge detection with particle swarm optimization, which improved feature-point localization accuracy. To reduce eccentricity errors caused by stripe width, Liao et al. [15] proposed a hollow-stripe stereoscopic target and combined it with the harmonic conjugate principle to achieve subpixel feature localization. In contrast, dynamic calibration methods rely on relative push-broom motion. They estimate parameters through continuous acquisition and stitching, usually in combination with planar calibration models. Hui et al. [16] proposed a quasi-dynamic calibration method based on an auxiliary area-scan camera and a dynamic electronic target, which reduced dependence on a high-precision constant-speed mechanical platform. Donné et al. [17] reconstructed the homography model and introduced bundle adjustment, thereby alleviating numerical instability caused by planar degenerate configurations and improving calibration accuracy and robustness under dynamic scanning conditions.

In recent years, the research focus has gradually shifted from single-camera geometric modeling to collaborative calibration of multi-line-scan systems and adaptation to complex application scenarios. For stereo and multi-camera collaborative calibration, Wang et al. [18] proposed a calibration method for stereo line-scan cameras based on a magnetic cylindrical-rod target, which enabled rapid estimation of intrinsic and extrinsic parameters for dual cameras. Zhang et al. [19] used a spherical target to perform hand–eye coordination and joint calibration for multiple line-scan laser cameras, thereby improving point-cloud registration accuracy. For special imaging configurations such as telecentric and panoramic line-scan systems, Lian et al. [20] established an enhanced imaging model with tilt-angle parameters and derived initial values using DLT, while Ait-Aider et al. [21] achieved decoupled estimation of intrinsic and mechanical parameters by using cross-ratio invariance and vanishing-point constraints. For large-scale multi-line-scan arrays and complex engineering applications, recent studies have further extended calibration from geometric modeling to system-level measurement collaboration and automated solving. Shi et al. [22] built a global calibration framework that integrates a laser tracker and hybrid targets. Andersen et al. [23] proposed a modular calibration board and combined it with deep learning to automate multidimensional parameter calibration for hyperspectral line-scan cameras. Zou et al. [24] introduced Bayesian optimization for refined extrinsic calibration in multi-sensor systems. Overall, related work has expanded from single-camera model estimation to multi-camera collaboration, complex configuration modeling, and automated calibration.

Existing studies have made substantial progress in geometric modeling, target design, and optimization-based parameter estimation, and many methods have been validated in a variety of industrial measurement scenarios. However, as measurement systems continue to develop toward higher precision, larger scale, and multi-sensor fusion, existing methods still have limitations in system simplification, stable parameter estimation, and globally consistent modeling. Therefore, current research on line-scan camera calibration still needs to address an important problem: how to reduce system complexity while maintaining calibration accuracy and how to improve robustness in complex environments.

To address the above issues, this paper establishes a unified imaging model and a target motion model for multi-line-scan camera systems. This paper introduces physical-scale and structural-prior constraints to anchor degenerate parameters, and formulates system calibration as a global joint optimization problem with physical constraints. In addition, this paper combines parameter normalization with a hierarchical optimization strategy to improve the numerical stability of the calibration process and the global consistency of the solution. Based on this design, this paper develops a high-precision calibration and three-dimensional reconstruction framework for multi-line-scan camera systems under complex dynamic operating conditions.

The remainder of this paper is organized as follows. Section 1 introduces the research background and related work on line-scan camera calibration and three-dimensional measurement. Section 2 establishes the measurement model of the multi-line-scan camera system and systematically analyzes the principles of calibration and reconstruction. Section 3 presents a globally consistent joint calibration algorithm for multi-line-scan cameras and provides the complete solution procedure. Section 4 validates the proposed method through simulations and experiments, and evaluates its overall performance in terms of accuracy, robustness, and stability. Section 5 concludes the paper.

## 2. System Model

### 2.1. Line-Scan Camera Imaging Model

Joint calibration and three-dimensional reconstruction of multi-line-scan camera systems require a unified imaging model and a unified geometric representation of measurement. According to the installation direction of the principal axis of the line-scan sensor in the camera coordinate system, this paper classifies the projection of line-scan cameras into two types: horizontal scanning and vertical scanning. On this basis, this paper presents a global imaging model for a multi-camera system under a unified world coordinate system.

**Figure 1:**
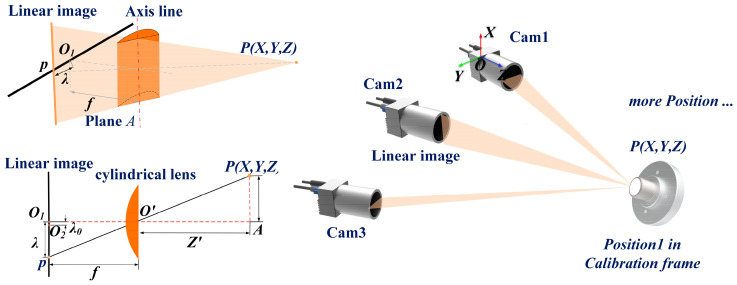
Principle of the multi-line-scan camera three-dimensional measurement system.

Figure 1 shows the structure of the multi-line-scan camera three-dimensional measurement system used in this paper. Let the coordinates of a spatial point in the camera coordinate system be *P*_c_ = [*X*,*Y*,*Z*]^T^, and let its projection on the one-dimensional sensor be the pixel coordinate λ. According to the installation direction of the line-scan sensor inside the camera, this paper divides the projection model of a line-scan camera into two basic forms.

For line-scan cameras whose sensor principal axis is parallel to the *X*-axis of the camera coordinate system (Cam1 and Cam3 in this system), the lateral displacement of a spatial point in the camera coordinate system determines its coordinate *λ_x_* on the line-scan camera. The projection relationship is expressed as follows:

(1) $$\lambda_{x}=f\cdot \frac{X}{Z}+c_{u}+\delta (x,z)$$

where *f* is the effective focal length, *X*, *Y*, *Z* are the three-dimensional coordinates of the target, *c_u_* is the principal-point coordinate of the sensor center, and *δ*(·) denotes the lens distortion residual.

For the line-scan camera with the sensor principal axis parallel to the Y-axis of the camera coordinate system (Cam2 in this system), the system acquires the projection information of the target in the vertical direction *λ_y_*, and the corresponding projection equation is given by:

(2) $$\lambda_{y}=f\cdot \frac{Y}{Z}+c_{u}+\delta (y,z)$$

The imaging model of a line-scan camera can be written in a unified form as a perspective projection from a three-dimensional spatial point to a one-dimensional pixel coordinate. This dimensionality-reduction projection from 3D to 1D makes the calibration process more prone to scale degeneracy and parameter coupling.

For the three-camera measurement system constructed in this paper, the world coordinate system *W* is defined to coincide completely with the camera coordinate system *C*_1_ of the reference camera Cam1. For the *i*-th camera (*i* = 2, 3), its pose with respect to the world coordinate system is jointly described by a rotation matrix *R_i_* ∈ *SO*(3) and a translation vector *T_i_* = [*T_x,i_*,*T_y,i_*,*T_z,i_*]^T^. This paper parameterizes rotation using a rotation vector. The rotation matrix *R*_i_ is computed from the rotation vector *r_i_* through the Rodrigues mapping (the transformation from a rotation vector to a rotation matrix), as follows:

(3) $$R_{i}=\mathrm{Rodrigues}(r_{i})$$

The coordinates of an arbitrary spatial point *P*_W_ in the coordinate system of the *i*-th camera can be expressed as follows:

(4) $$P_{c,i}=R_{i}^{⊤}(P_{w}-T_{i})$$

Substituting Equation (4) into the corresponding projection model, i.e., Equation (1) or Equation (2), yields the global projection expression of the spatial point on the image plane of an arbitrary line-scan camera. This expression enables geometric modeling of multi-camera observation data under a unified world coordinate system and provides a unified framework for subsequent multi-view joint adjustment and system-level calibration.

### 2.2. Calibration Target Kinematic Model

This paper uses an H-shaped planar target with four feature points *P_j_* (*j* = 1,…4) as the calibration object. The coordinates of these feature points in the target coordinate system *L*, denoted by *P_Lj_*, are known constants and provide stable structural geometric constraints. Assume that *N* frames of observations are collected during dynamic calibration. For the *k*-th frame (*k* = 1,…*N*), the pose of the target with respect to the world coordinate system *W* is described by the rotation vector *r*_rig,*k*_ and the translation vector *t*_rig,*k*_. Then, the spatial position of the *j*-th feature point coordinate *P_w_*_,*j*,*k*_ in the world coordinate system can be expressed as follows:

(5) $$P_{w,j,k}=r_{\mathrm{rig},k}P_{L,j}+t_{\mathrm{rig},k}$$

Equation (5) gives a unified parametric expression of the target feature points over the time sequence, allowing multi-frame observations to be associated through the same set of system parameters and frame-wise pose parameters. Compared with independent modeling for each frame, this kinematic description introduces additional geometric constraints along the temporal dimension. These constraints help alleviate parameter degeneracy in line-scan camera calibration and provide a consistent parameter space for subsequent joint optimization.

The known fixed distance between two feature points in the target structure is denoted by *D*_ref_. The fixed distance between two feature points, for example, the first point *P_w_*_,1,*k*_ and the second point *P_w_*_,2,*k*_, satisfies the following condition in the *k*-th frame:

(6) $$|P_{w,1,k}-P_{w,2,k}|=D_{\mathrm{ref}}$$

This uniqueness constraint restricts the reconstructed results of all frames to a consistent physical scale and reduces the coupling between the focal length and depth-related parameters.

When constructing the parameter set for joint optimization, this paper screens parameter observability according to the specific imaging configuration. For the line-scan camera Cam2 in the vertical-scanning configuration, the imaging model is insensitive to translation along the *X*_W_ direction of the world coordinate system. The partial derivative of the reprojected coordinate *u*_y_ with respect to the lateral translation parameter *T_x_*_,2_ satisfies the following:

(7) $$\frac{\partial u_{y}}{\partial T_{x,2}}=\frac{\partial u_{y}}{\partial y_{c}}\cdot \frac{\partial y_{c}}{\partial T_{x,2}}\approx 0$$

This result indicates that *T_x_*_,2_ is unobservable under the current observation configuration. If it is optimized together with the other parameters, it may cause rank deficiency in the Jacobian matrix or worsen the numerical conditioning, thereby reducing overall convergence stability. Therefore, *T_x_*_,2_ is fixed during parameter estimation and is not included as an optimization variable. Because the vertical line-scan imaging geometry is naturally decoupled from translation in this direction, the value of *T_x_*_,2_ does not change the error distribution of the objective function and does not materially affect the convergence of the other observable parameters or the final three-dimensional reconstruction accuracy.

In summary, the model introduces target kinematic constraints, physical-scale constraints, and an unobservable-parameter handling strategy at the same time. These designs improve parameter identifiability and numerical stability within a unified geometric framework, and provide the constraint basis for the subsequent development of the global joint calibration and three-dimensional reconstruction algorithm.

## 3. Global Calibration Algorithm and Solution

### 3.1. Calibration Objective Function Formulation

**Figure 2:**
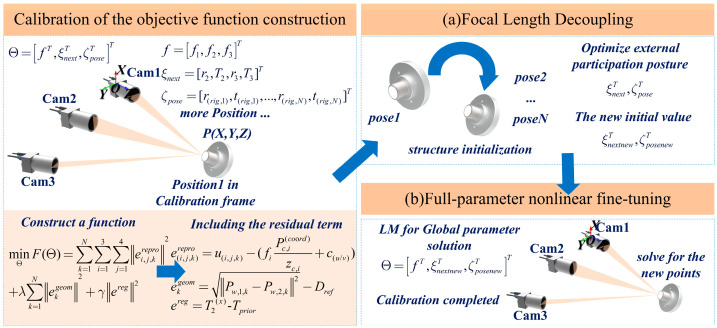
Flowchart of the algorithm.

Building on the multi-line-scan camera imaging model and parameter observability analysis established in Section 2, this paper develops a system-level global calibration optimization framework, and the overall algorithm flow is shown in Figure 2.

To address the common issues in multi-line-scan camera systems, including strong parameter coupling, scale ambiguity, and a highly nonconvex solution space, this paper proposes a nonlinear global bundle adjustment method that integrates geometric-constraint anchoring and scale normalization. The method explicitly introduces physical-scale constraints to suppress parameter degeneracy, and adopts a staged convergence strategy to improve numerical stability and overall convergence reliability under large-deviation initial values.

The system-level global parameter vector to be optimized is denoted by Θ, and consists of the camera intrinsic parameters, camera extrinsic parameters, and the motion poses of the calibration target over multiple sampled frames:

(8) $$\Theta ={\left[f^{⊤},\xi_{\mathrm{ext}}^{⊤},\zeta_{\mathrm{poses}}^{⊤}\right]}^{⊤}$$

where *f* = [*f*_1_,*f*_2_,*f*_3_]^T^ denotes the effective focal lengths of the three cameras; *ξ*_ext_ = [r_2_,T_2_,r_3_,T_3_]^T^ denotes the extrinsic parameters of Camera 2 and Camera 3 with respect to the reference camera; and *ζ*_poses_ = [r_rig,1_,t_rig,1_,…,r_rig,N_,t_rig,N_]^T^ denotes the six-degree-of-freedom poses of the target over N sampled frames.

Under multi-frame and multi-camera observations, the calibration problem can be formulated as a joint estimation of the parameter vector Θ. The objective is to minimize the deviation between the observed pixel coordinates and the model predictions while satisfying the geometric consistency constraints of the system. By incorporating the physical-scale constraints and parameter observability analysis in Section 2, this paper constructs a joint objective function composed of a reprojection error term, a geometric consistency constraint term, and a structural regularization term:

(9) $$\underset{\Theta }{\min}F(\Theta )=\sum_{k=1}^{N}\sum_{i=1}^{3}\sum_{j=1}^{4}\|e_{i,j,k}^{\mathrm{repro}}{\|}^{2}+\lambda \sum_{k=1}^{N}\|e_{k}^{\mathrm{geom}}{\|}^{2}+\gamma \|e^{\mathrm{reg}}{\|}^{2}$$

where *λ* and *γ* are the weighting coefficients of the geometric constraint term and the regularization term, respectively.

The reprojection residual is used to measure the deviation between the predicted pixel position of a spatial point under the imaging model and the actual observation, and is defined as follows:

(10) $$e_{i,j,k}^{\mathrm{repro}}=u_{i,j,k}-\left(f_{i}\cdot \frac{P_{c,i}^{\left(\mathrm{coord}\right)}}{z_{c,i}}+c_{u/v}\right)$$

where *u*_i,j,k_ denotes the actual observed pixel coordinate of the *j*-th target feature point in the *k*-th frame on the *i*-th camera, and $P_{\left(c,i\right)}^{\left(coord\right)}$ takes the *X* or *Y* component according to the scanning direction of the camera.

The geometric consistency constraint term introduces the known physical distances between target feature points, thereby suppressing scale degeneracy in the line-scan imaging model. For the k-th frame, the corresponding constraint residual is written as follows:

(11) $$e_{k}^{\mathrm{geom}}=\sqrt{{\left\|P_{\left(w,1,k\right)}-P_{\left(w,2,k\right)}\right\|}^{2}}-D_{\mathrm{ref}}$$

The structural regularization term corresponds to the weakly observable or unobservable directions identified in the observability analysis. To address the unobservability of the lateral translation parameter in the vertically scanning line-scan camera, this paper imposes prior constraints on the related parameter to suppress drift along singular directions. The corresponding residual term is written as follows:

(12) $$e^{\mathrm{reg}}=T_{2}^{\left(x\right)}-T_{\mathrm{prior}}$$

The joint objective function formed by Equations (9)–(12) preserves consistency with the observation data while incorporating physical-scale information and structural priors into a unified optimization framework, thereby improving the stability and global consistency of multi-line-scan camera system calibration.

### 3.2. Numerical Optimization and Staged Convergence Strategy

We adopt a Two-stage optimization based on the Levenberg–Marquardt (LM) algorithm to solve the joint objective function defined by Equations (9)–(12). The variables to be optimized include the focal length parameters, the camera extrinsic parameters, and the multi-frame target pose parameters. Since the rotation, translation, and focal length parameters differ in numerical scale, parameter update scaling is introduced during optimization to improve the stability of the iterative process. In this strategy, scaling is mainly applied to the camera rotation parameters and the target rotation parameters in order to limit their iteration step sizes at the early stage, while the focal length and translation parameters are kept at their original scales or optimized according to their physical units.

In the specific solution process, a two-stage optimization based on the Levenberg–Marquardt algorithm is employed. In the first stage, the initial focal length is fixed, and only the camera extrinsic parameters and the multi-frame target poses are optimized, so that the relative camera configuration and the spatial structure of the target can first converge to a stable state. In the second stage, the focal length parameters are released based on the results of the first stage, and full-parameter joint optimization is then carried out to achieve consistent convergence of the focal length, extrinsic parameters, and target pose parameters. This algorithm does not alter the original form of the objective function, but improves the convergence stability of joint calibration through parameter scaling and staged optimization at the solver level. The detailed procedure is given in Algorithm 1.
**Algorithm 1** Two-stage optimization based on the LM algorithm**CALIBRATE** Θ = [*f*,*ξ*_ext_,*ζ*_poses_] **STAGE 1** Set Θ_1_ ← [$\xi_{ext}^{\left(0\right)}$,$\xi_{poses}^{\left(0\right)}$] Set scaling vector S_1_ for rotation parameters **repeat** compute residual r_1_(*f*^(0)^,*ξ*_ext_,*ζ*_poses_) compute Jacobian *J*_1_ solve ($J_{1}^{T}$*J*_1_ + *u*I)ΔΘ_1_ = −$J_{1}^{T}$*r*_1_ update Θ_1_←Θ_1_ + ΔΘ_1_ adjust *u* **until** convergence **STAGE 2** set Θ_2_ ← [*f*^(0)^,Θ_1_] Set scaling vector S_2_ for rotation parameters **repeat** compute residual r_2_(*f*,*ξ*_ext_,*ζ*_poses_) compute Jacobian *J*_2_ solve ($J_{2}^{T}$*J*_2_ + *u*I)ΔΘ_2_ = −$J_{2}^{T}$*r*_2_ update Θ_2_←Θ_2_ + ΔΘ_2_ adjust *u* **until** convergence **return** Θ^∗^ = Θ_2_

In summary, the above two-stage optimization algorithm, combined with the joint objective function, alleviates the strong parameter coupling and numerical instability in line-scan camera calibration through parameter scale adjustment and staged optimization, thereby significantly improving the stability and reliability of joint parameter estimation. In the next chapter, simulation experiments will be conducted to evaluate and analyze the convergence performance, calibration accuracy, and three-dimensional reconstruction results of the proposed algorithm.

## 4. Experiments and Results Discussion

### 4.1. Simulation Setup and Evaluation Metrics

This paper constructs a high-fidelity line-scan camera simulation measurement environment to generate simulated observation data with explicit physical ground truth. The simulation system is designed to closely match industrial-grade line-scan camera measurement systems in geometric structure, imaging model, and noise characteristics, thereby ensuring the credibility and reproducibility of simulation results for calibration accuracy evaluation. The simulation measurement system consists of three line-scan cameras. Its core imaging parameters, installation parameters, and scanning modes are configured according to typical industrial application scenarios. The detailed parameter settings are listed in Table 1.

In terms of geometric configuration, Cam1 serves as the reference camera of the system, and its camera coordinate system is defined as the origin of the global coordinate system. Cam2 and Cam3 are arranged in different directions to enhance spatial constraint capability. Specifically, Cam2 uses the vertical scanning mode, and its optical axis is translated by approximately 400 mm along the X-direction relative to Cam1. Cam3 uses the horizontal scanning mode, with a baseline span of approximately 800 mm.

The generation of simulated data strictly follows the real physical motion model. The calibration target undergoes random six-degree-of-freedom rigid-body motion within a depth range of 1200 mm to 3200 mm from the cameras. To enhance imaging parallax and reduce the risk of parameter coupling, each target pose includes large attitude variations. The pitch and yaw angles are both set within $\pm 23^{\circ }$ (approximately 0.4 rad), so that the observation sequence can effectively decouple key parameters such as focal length, baseline distance, and depth.

Zero-mean Gaussian white noise is added to the ideal projected pixel positions to simulate random errors introduced by subpixel edge extraction or center localization algorithms in actual line-scan camera measurement. On this basis, this paper constructs the simulated observation model, which is expressed as follows:

(13) $$u_{ijk}=u\left(\Theta ,P_{w,j,k}\right)+\varepsilon ,\;\varepsilon \sim N(0,\sigma^{2})$$

where *u*(·) denotes the theoretical projection function based on the true intrinsic and extrinsic parameters, and σ denotes the observation noise level, which is set to 0.3 pixel by default to represent the statistical error level of high-precision subpixel feature extraction algorithms.

This paper establishes an evaluation metric system that includes fitting accuracy, parameter recovery capability, and spatial measurement accuracy. By computing the absolute deviations between the estimated values of key parameters, such as the focal length *f*, camera baseline *T_x_*, and installation height *T_z_*, and their physical ground-truth values, this paper evaluates the ability of the algorithm to recover true physical parameters:

(14) $$\Delta P=\left|P_{\mathrm{estimated}}-P_{\mathrm{true}}\right|$$

After calibration, the estimated camera intrinsic and extrinsic parameters are used to triangulate random test points in the measurement field. This paper then computes the Euclidean distance between each reconstructed point *P*_est_ and its theoretical ground-truth point *P*_gt_ to evaluate the effective accuracy of the system in practical three-dimensional measurement tasks:

(15) $$E_{3D}=\left\|P_{\mathrm{est}}-P_{\mathrm{gt}}\right\|$$

Based on the above high-fidelity simulation measurement environment and the evaluation metric system, this chapter systematically analyzes and comparatively validates the performance of the proposed global calibration algorithm from three aspects: reprojection error, key-parameter recovery accuracy, and three-dimensional measurement error.

### 4.2. Comprehensive Experimental Analysis of Initialization Parameters

**Figure 3:**
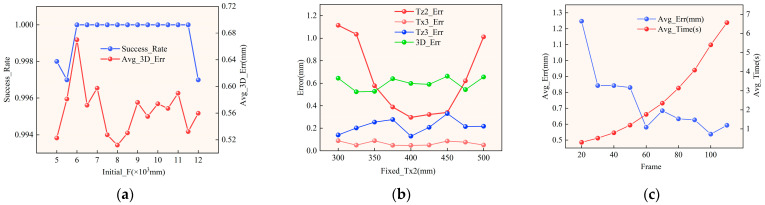
Influence of initialization-related factors on parameter convergence and 3D measurement accuracy: (**a**) influence of the initial focal length on the convergence success rate and 3D measurement error; (**b**) influence of the initial value of $T_{x2}$ on selected extrinsic parameters and 3D measurement error; (**c**) influence of the number of iterative frames on 3D measurement error and computation time.

This paper conducts a systematic experimental analysis of three key factors, namely initial focal-length perturbation, cross-camera baseline physical-prior constraints, and the number of effective motion frames, to systematically evaluate the numerical stability, parameter robustness, and computational efficiency of the proposed global calibration and three-dimensional reconstruction algorithm under complex engineering conditions. The experiments also comprehensively examine how these factors affect parameter convergence behavior and three-dimensional measurement accuracy. The results are shown in Figure 3.

First, to evaluate the sensitivity of the system to uncertainty in the initial intrinsic parameters, this paper systematically perturbs the initial focal length within the range of [5000, 12,000] pixels, and records the calibration convergence success rate and the mean three-dimensional reconstruction error under different initial values. The results are shown in Figure 3a. Across the entire test range, the algorithm maintains a very high convergence success probability. The success rate is never lower than 99.7% and reaches 100% over most of the range. These results indicate that the proposed multi-stage optimization strategy can effectively avoid local minima and divergence, and thus provides excellent global convergence capability.

From the perspective of three-dimensional reconstruction accuracy, as the initial focal length increases from 5000 pixels to 12,000 pixels, the reconstruction error remains stable within the range of 0.51–0.67 mm. The maximum error is 0.6698 mm, and the minimum error is 0.5118 mm. The fluctuation amplitude is less than 0.16 mm, and no significant deterioration is observed as the deviation of the initial value increases. In particular, when the initial focal length lies in the range of 7000–10,000 pixels, the reconstruction error remains stably within 0.53–0.60 mm for most cases, showing a clear plateau behavior. These results demonstrate that the proposed algorithm is strongly insensitive to the initial intrinsic parameters and can still converge stably to a high-accuracy solution under large-range perturbations of the initial values.

Second, with all other parameters fixed, this paper sets *T_x_*_2_ to values from 300 mm to 500 mm and analyzes the resulting variations in extrinsic parameter estimation errors and three-dimensional reconstruction error, in order to systematically evaluate the effect of the physical prior constraint on the cross-camera baseline parameter *T_x_*_2_ on system geometric stability. The results are shown in Figure 3b. The experimental results show that variations in *T_x_*_2_ have a certain influence on the estimation accuracy of the extrinsic-parameter components, especially in the significant fluctuation of the depth-direction error *T*_z2_ of Cam2. When *T_x_*_2_ deviates from the true mechanical installation value, the error of *T*_z2_ increases noticeably. However, the corresponding three-dimensional reconstruction error remains within the range of 0.52–0.66 mm overall. It does not show a monotonic trend with changes in *T_x_*_2_, nor does it exhibit a clearly optimal baseline configuration. These results indicate that the proposed joint calibration and three-dimensional reconstruction model is insensitive to the initial setting of the cross-camera baseline parameter *T_x_*_2_. The method can still converge stably to a consistent solution under a relatively large range of initial-value deviations, demonstrating good convergence consistency and numerical stability of the system optimization framework and thereby ensuring the reliability of three-dimensional measurement results.

Third, this paper analyzes frame efficiency to evaluate the balance achieved by the proposed method between accuracy and computational complexity. By gradually increasing the number of effective frames involved in optimization, this paper records the changes in three-dimensional reconstruction error and total computation time under different frame counts. The results are shown in Figure 3c. The experimental results show that when the number of frames increases from 20 to 60, the system reconstruction error decreases rapidly from 1.247 mm to 0.581 mm, with an error reduction of more than 53%. This result indicates that, in the low-frame regime, introducing motion redundancy information can significantly enhance the strength of geometric constraints in the system. As the number of frames further increases to the range of 80–110, the reconstruction error gradually becomes stable, with the overall fluctuation controlled within 0.53–0.63 mm, showing a clear convergence plateau behavior.

At the same time, the computation time increases approximately linearly, from 0.298 s at 20 frames to 6.568 s at 110 frames, which verifies that the proposed algorithm maintains good computational controllability while preserving high accuracy. Considering both accuracy and efficiency, the results show that when the number of frames is in the range of 60–80, the system can achieve sub-millimeter three-dimensional reconstruction accuracy with a computation cost of 2–3 s, thus providing a good balance among accuracy, efficiency, and stability. This finding provides a practical basis for selecting engineering parameters in subsequent online calibration and dynamic measurement applications.

Finally, in the comparative simulation experiments, multiple representative discrete points within the focal length range of [5000, 12,000] were selected as initial inputs, and the proposed method was compared with the classical bundle adjustment method and an ablation model. The classical bundle adjustment (Classic BA) method directly applies the LM algorithm to perform one-shot joint optimization of the focal length parameters, camera extrinsic parameters, and multi-frame target pose parameters under the same objective-function framework. The ablation method (Ablation) retains the same two-stage optimization procedure as the proposed method, but removes parameter scaling, that is, all parameters participate directly in the Levenberg–Marquardt iterations using their original numerical scales. The evaluation metrics include convergence rate, three-dimensional reconstruction accuracy, and image fitting residual, with comparisons conducted for focal length initial values of 5000 and 10,000. The representative quantitative comparison results for the different methods are shown in Table 2.

Table 2 presents a detailed comparison of the algorithm performance under different initialization conditions. When the initial value deviates significantly from the ground truth, with f = 5000, the traditional Classic BA exhibits severe ill-conditioning, with a convergence rate of only 2.2%, and fails to produce valid reconstruction accuracy, reported as N/A. In contrast, the proposed method maintains extremely high robustness under the same condition, achieving a convergence rate of 99.8%, while its 3D reconstruction error of 0.531 mm and fitting residual of 0.230 pixel are both significantly better than those of Ablation. When the initial value lies within an ideal range, with f = 10,000, although all methods achieve relatively high convergence rates, the proposed method still maintains leading or comparable performance across all evaluation metrics. These results strongly demonstrate that the proposed algorithm effectively overcomes the local minimum problem and greatly enlarges the convergence basin of the solver.

In summary, the proposed method is systematically validated from three aspects, namely robustness to initial parameter perturbation, structural physical prior constraints, and frame efficiency. The experimental results show that the method is able to maintain stable convergence and high-precision reconstruction performance under large initial perturbations, structural scale uncertainty, and limited observation frames. Meanwhile, a horizontal comparison among different methods further indicates that the two-stage optimization method with parameter scaling achieves the best robustness and solution accuracy.

### 4.3. Calibration and Measurement Error Analysis

**Figure 4:**
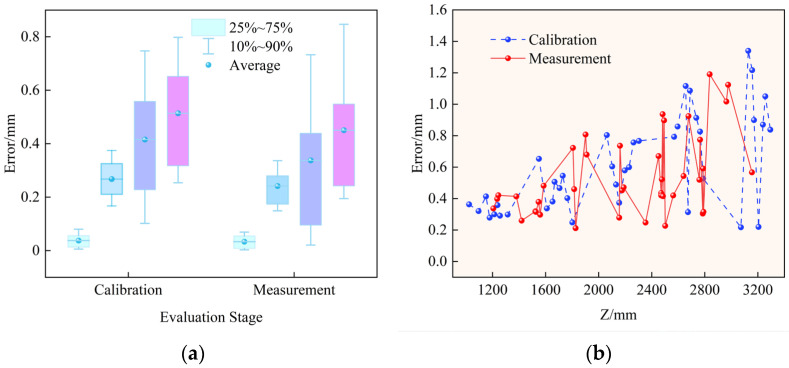
Relationship between 3D error distributions in the calibration and measurement stages: (**a**) Statistics of the *X*, *Y*, *Z*, and overall 3D errors during the calibration and measurement stages; for each method, the results are presented from left to right as X, Y, Z, and overall 3D error.; (**b**) 3D errors versus depth for the calibration and measurement stages.

This paper statistically analyzes the distribution of three-dimensional reconstruction errors in both the calibration stage and the measurement stage using the simulation system, and systematically evaluates the geometric consistency and spatial error propagation characteristics of the proposed global calibration algorithm in practical measurement tasks. In the calibration set, 60 positions are generated, and 40 of them are selected for three-dimensional reconstruction. Subsequently, another 40 points are selected within the calibration field range as the measurement set. On this basis, this paper analyzes how the three-dimensional error evolves with target depth. The corresponding experimental results are shown in Figure 4.

First, from the perspective of overall error distribution characteristics, the statistical results of the three-dimensional errors in the calibration stage and the measurement stage show high consistency, as illustrated in Figure 4a. For the 40 groups of spatial point samples, the 3D errors in the calibration stage are mainly concentrated in the range of 0.2–0.9 mm, with a mean error of approximately 0.56 mm and a maximum error of about 1.20 mm. The 3D error range in the measurement stage highly overlaps with that in the calibration stage, with a mean error of approximately 0.54 mm and a maximum error of about 1.32 mm. The two stages remain highly consistent in terms of mean, variance, and extreme-value range. This result indicates that, after parameter estimation, the proposed global calibration model can be stably transferred to practical measurement tasks, without obvious overfitting or geometric-structure degradation.

From the perspective of directional error components, the distribution proportions of the 3D error along the *X*, *Y*, and *Z* directions are basically consistent in both the calibration and measurement stages. Among them, the Z-direction error is dominant, followed by the Y-direction error, while the X-direction error is the smallest. This result indicates that the proposed multi-stage calibration strategy achieves stable coupled modeling of intrinsic and extrinsic parameters during optimization, thereby ensuring stable three-dimensional geometric consistency in both the calibration and measurement stages.

Second, this paper performs a systematic statistical analysis of the relationship between three-dimensional reconstruction error and target depth. The results are shown in Figure 4b. In both the calibration stage and the measurement stage, the 3D error shows a highly consistent evolution trend as depth changes. In the near-field region (Z < 1800 mm), the system 3D error remains stably within 0.2–0.5 mm, and the error increases slowly, indicating that the system provides very high spatial resolution under close-range operating conditions. As the target depth increases to the mid-range interval (1800–2600 mm), the error gradually increases and is mainly concentrated in the range of 0.4–0.9 mm, showing an overall near-linear upward trend. In the far-field region (Z > 2600 mm), the error of some points increases to 1.0–1.8 mm, mainly due to the geometric magnification effect caused by the depth resolution degrading approximately with the square of distance. Across the entire working-distance range, the error curves of the calibration stage and the measurement stage remain highly consistent in both magnitude and trend, and no systematic shift or abrupt error change is observed.

The above results show that, within the typical industrial working-distance range of 1.0–3.2 m, the overall three-dimensional measurement error of the proposed system remains stably below 1 mm. In addition, the error distribution varies with depth in a continuous and predictable manner, meeting the stringent requirements for stability and reliability in large-scale, high-precision industrial measurement scenarios.

### 4.4. Noise Error Analysis

**Figure 5:**
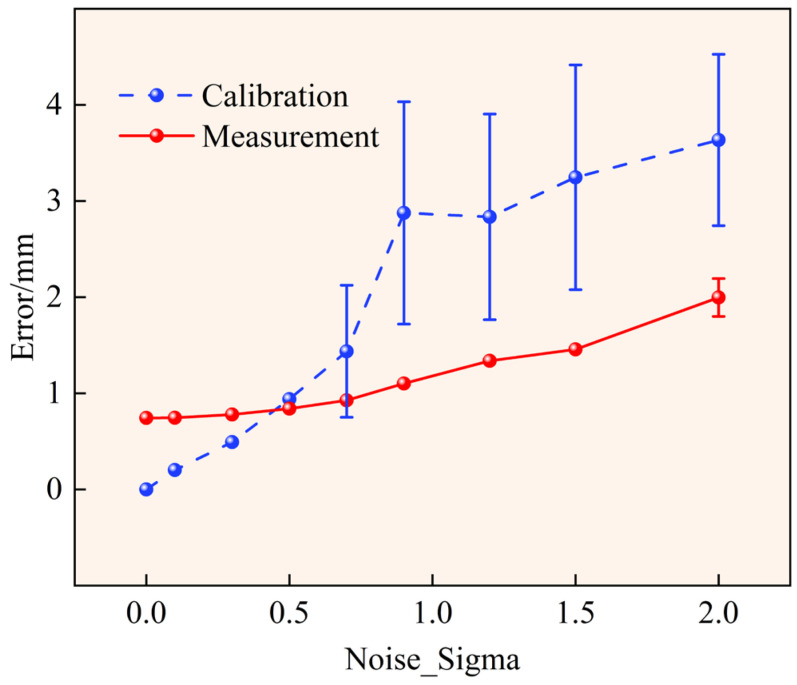
Comparison of three-dimensional reconstruction errors under added noise.

In the simulation environment, this paper gradually increases the standard deviation σ of the observation Gaussian noise and performs repeated experiments on the system calibration process and three-dimensional reconstruction results to evaluate the influence of imaging noise on the calibration accuracy and 3D measurement accuracy of the multi-line-scan measurement system. The noise standard deviation σ takes several typical values in the range of 0–2, including the noise-free condition (σ = 0). The calibration error statistics correspond to the calibration results obtained under each noise level. For the 3D measurement error statistics, this paper uses the system parameters obtained by calibration at σ = 0.3 as fixed calibration inputs, and then performs repeated measurement experiments under different measurement noise levels. Multiple independent trials are conducted at each noise level, and the means and standard deviations of the calibration error and measurement error are computed separately. The results are shown in Figure 5. The statistics show that, in the range of σ ≤ 0.5, the standard deviations of both calibration error and measurement error are close to zero, and the fluctuations are small; therefore, the error bars for this range are not plotted separately in the figure.

From the calibration error results, the system is relatively sensitive to imaging-noise perturbations. When σ = 0, the calibration error is 0, indicating that under noise-free simulation conditions, the established imaging model and the global calibration solution process can accurately recover the system parameters, which verifies the effectiveness of the modeling and solution pipeline. As the noise level increases, the calibration error increases overall. In the low-noise interval (σ = 0~0.5), the calibration error gradually increases from 0 to 0.9388, with a relatively smooth trend. When the noise level increases to σ = 0.7 and σ = 0.9, the calibration error reaches 1.4367 and 2.876, respectively, while the standard deviation increases to 0.6865 and 1.1557, indicating a clear increase in the dispersion of the calibration solutions. In the higher-noise range, the calibration error remains at a relatively high level with some fluctuations (e.g., 2.8358 at σ = 1.2, 3.247 at σ = 1.5, and 3.6348 at σ = 2), indicating that the stability of parameter estimation decreases under high-noise conditions.

The statistical conditions for the 3D measurement error differ from those for the calibration error, because the calibration parameters are fixed to the calibration results obtained under the noise condition of σ=0.3. The results show that the measurement error increases gradually as the noise level increases, but the trend is much smoother than that of the calibration error. Under the noise-free measurement condition (σ = 0), the measurement error is 0.7442. In the low-noise interval (σ = 0.1~0.5), the measurement error increases from 0.745 to 0.8407, showing only a small overall change. When σ = 0.7 and σ = 0.9, the measurement error is 0.9274 and 1.1015, respectively, and continues to increase smoothly. As the noise level further increases, the measurement error continues to rise (1.3382 at σ = 1.2, 1.4571 at σ = 1.5, and 1.9971 at σ = 2), but remains lower than the corresponding calibration error at all noise levels. In terms of standard deviation, except for the measurement-error standard deviation of 0.1966 at σ = 2, the standard deviations at the other noise levels are all close to zero. This result indicates that, under the current experimental settings, the reconstruction results exhibit small overall fluctuations and good stability.

Overall, imaging noise affects calibration-parameter estimation more directly, and under medium-to-high noise conditions, it clearly leads to increases in both error and fluctuation. In contrast, with fixed calibration parameters, the influence of measurement-stage noise on 3D reconstruction error grows more gradually. These results indicate that the proposed geometric constraint model and joint solution method can maintain relatively stable 3D reconstruction performance under noise perturbations and exhibit a certain degree of error suppression capability.

### 4.5. Experimental Results and Discussion

**Figure 6:**
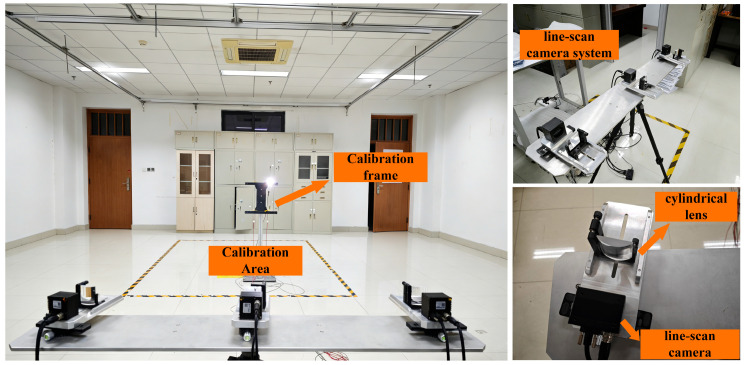
Experimental scene and line-scan system.

A real measurement platform consisting of the multi-line-scan camera system and an I-shaped calibration target was established in this study, and corresponding measurement experiments were carried out. The multi-line-scan camera system was used to acquire target images and perform feature extraction as well as spatial coordinate reconstruction, while the I-shaped calibration target served as the measured object for evaluating the capability of the system in recovering geometric structure and scale information. By constructing the measurement platform under real experimental conditions, the spatial reconstruction capability and dimensional measurement performance of the proposed method can be validated. The experimental platform and measurement scene are shown in Figure 6.

In the experiment, the multi-line-scan camera system was first calibrated to establish the intrinsic and extrinsic parameter relationships of the cameras and to impose global constraints under a unified coordinate reference. Subsequently, the I-shaped calibration target was moved within the measurement area of the real experimental scene to acquire image data, and the three-dimensional coordinates of the target feature points were reconstructed using the feature extraction and matching method proposed in this paper. Based on these reconstructed points, the distances between key feature points on the calibration target were further calculated. To ensure the rationality and comparability of the experimental analysis, geometric quantities that are insensitive to changes in the coordinate system were selected as evaluation metrics, namely the distances between feature points and the measured rod lengths.

Considering that the I-shaped calibration target has clear structural characteristics and stable geometric dimensions, its reconstruction results can not only reflect the capability of the system to recover the overall spatial shape of the target, but also intuitively reveal the spatial distribution of measurement errors across different frames. Therefore, after completing the analysis of distance measurement and scale recovery, the three-dimensional reconstruction results of all frames after global calibration were further visualized together with the camera positions. In Figure 6, the true structure of the I-shaped calibration target is taken as the reference, and the reconstructed spatial positions of all frames are restored accordingly. At the same time, different measurement frames are color-coded according to the joint comprehensive error of the entire calibration target, so as to analyze the spatial recovery performance of the system and the error distribution characteristics under real experimental conditions. For visualization purposes, the camera positions were mapped to a closer distance according to a proportional relationship, while the actual coordinate axis values were established with the left camera as the coordinate origin.

**Figure 7:**
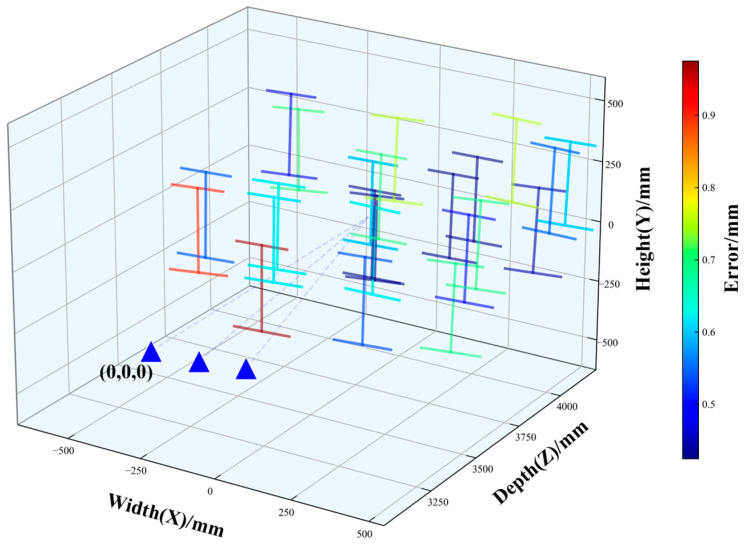
Calibration error and reconstruction of the calibration target. The three blue triangles represent the position (virtual) of the line array camera, which is mapped to the near distance through dotted lines.

As shown in Figure 7, the reconstructed I-shaped calibration target is highly consistent with the true calibration target in its overall shape. The reconstructed results of different frames can preserve the geometric contour and relative spatial relationships of the target well, indicating that the proposed global calibration method is able to effectively recover the three-dimensional spatial structure of the target. According to the error statistics, when 24 frames of the calibration target were used for calibration, the average reconstruction accuracy of the system reached 0.61 mm. Meanwhile, the maximum joint comprehensive error was 0.9 mm, the minimum was 0.42 mm, and the standard deviation was 0.15 mm, showing that the overall error distribution among frames is relatively concentrated. These results demonstrate that the proposed method achieves good overall accuracy in the global calibration task.

Since Figure 7 is plotted based on the joint results, the color variation reflects the overall reconstruction quality of the entire I-shaped calibration target rather than the variation in a single dimensional indicator. From the statistics of individual error components, the maximum, minimum, and standard deviation of the top-width error are 1.27 mm, 0.042 mm, and 0.43 mm, respectively, whereas those of the bottom-width error are 1.14 mm, 0.16 mm, and 0.32 mm, respectively. The comparison indicates that the top-width error shows slightly greater dispersion than the bottom-width error, suggesting that the upper part of the calibration target is more strongly affected by imaging conditions, feature extraction, and matching stability during reconstruction, while the reconstruction of the lower part is relatively more stable.

Overall, although the reconstruction accuracy varies to some extent under different observation viewpoints, the spatial position and shape of the I-shaped calibration target can still be recovered stably in most cases. This indicates that the proposed three-dimensional reconstruction method based on global calibration has a certain adaptability to viewpoint variation. The spatial distribution of the joint comprehensive error shown in Figure 7 is consistent with the statistical results of the top-width and bottom-width errors. The reconstructed results are generally clustered around the reference calibration target, and the small standard deviation of the joint error further verifies the effectiveness of the method. However, the differences in error among different structural parts also suggest that the robustness of the current method under complex observation conditions still has room for further improvement.

## 5. Conclusions

To address parameter coupling, scale ambiguity, and numerical instability in multi-line-scan camera system calibration, this paper investigates geometric modeling, constraint construction, and optimization-based solution strategies and develops a global calibration and three-dimensional reconstruction method for complex dynamic and multi-camera collaborative scenarios. Based on unified imaging and motion models, the method introduces physical-scale constraints to compensate for parameter degeneracy and combines them with a staged convergence strategy to improve solution stability and convergence reliability under large initial-value deviations. Simulation results show that, when the initial focal length is perturbed within 5000–12,000 pixels, the algorithm maintains a convergence success rate above 99.7%, while the 3D reconstruction error remains stable within 0.51–0.67 mm. When the cross-camera baseline parameter *T_x_*_2_ deviates from the true value within 300–500 mm, the reconstruction error remains within 0.52–0.66 mm, indicating good adaptability to the initial baseline setting. The frame-count analysis shows that sub-millimeter reconstruction accuracy (0.53–0.63 mm) can be achieved using 60–80 observation frames, while the computation time remains approximately 2–3 s. Noise-perturbation experiments further show that, under strong noise conditions (σ = 2), the measurement error is still controlled within 1.87 mm. Although the calibration error increases with noise level, the geometric constraint model can suppress, to a certain extent, error propagation to the final measurement results. Overall, the experimental results demonstrate that the proposed method maintains stable convergence and achieves high-accuracy 3D measurement under conditions of large initial-value deviations, limited observation frames, and noise interference. These results indicate that the proposed method can provide reliable technical support for high-precision calibration and online measurement applications of multi-line-scan camera systems in engineering scenarios.

## Figures and Tables

**Table 1:** Simulation parameter settings for the line-scan measurement system.

Category	Parameter	Symbol	Ground Truth
Camera intrinsics	Effective focal length	*f*_1_, *f*_2_, *f*_3_	9005.0, 9148.0, 9052.0 (pixel)
	Principal point coordinates	*c_u_*, *c_v_*	2048.0, 2048.0 (pixel)
	Sensor resolution	Res	4096 × 1 (pixel)
System extrinsics	Relative pose of Cam2	[*r*_2_∣*T*_2_]	[0.015, −0.01, 0.0] rad, [400.5, 0, 10.2] mm
	Relative pose of Cam3	[*r*_3_∣*T*_3_]	[0, 0.02, −0.01] rad, [799.1, 0, −14.8] mm
Calibration target	Feature-point distance	*D*_ref_	500.00 mm (*P*_1_–*P*_2_)
	Target height	*H*_rig_	300.00 mm (*P*_1_–*P*_3_)
Sampling settings	Number of dynamic sampling frames	*N*	60 frames(default)
	Working distance range	*Z*_work_	1200 mm∼3200 mm
	Calibration angle range	*θ*, *ϕ*, *ψ*	±0.4 rad (≈±23°)
Random disturbance	Image observation noise	*σ*	0.3 pixel (default)

**Table 2:** Comparison of results from different calibration methods.

Method	Rate of Convergence (5 k/10 k)	3D Err (mm) (5 k/10 k)	RMSE (Pixel) (5 k/10 k)
proposed	99.8%/100%	0.531/0.556	0.230/0.207
Ablation	12.5%/30.2%	2.436/0.883	0.774/0.310
Classic BA	2.2%/99.8%	(N/A)/0.596	(N/A)/0.215

## Data Availability

Data are contained within the article.
